# Prognostic impact of incident left ventricular systolic dysfunction after myocardial infarction

**DOI:** 10.3389/fcvm.2022.1009691

**Published:** 2022-09-29

**Authors:** Goro Yoshioka, Atsushi Tanaka, Nozomi Watanabe, Kensaku Nishihira, Masahiro Natsuaki, Atsushi Kawaguchi, Yoshisato Shibata, Koichi Node

**Affiliations:** ^1^Department of Cardiovascular Medicine, Saga University, Saga, Japan; ^2^Department of Cardiovascular Physiology, Faculty of Medicine, University of Miyazaki, Miyazaki, Japan; ^3^Miyazaki Medical Association Hospital Cardiovascular Center, Miyazaki, Japan; ^4^Center for Comprehensive Community Medicine, Saga University, Saga, Japan

**Keywords:** acute myocardial infarction, left ventricular ejection fraction, left ventricular systolic dysfunction, prognosis, reassessment

## Abstract

**Introduction:**

We sought to investigate the prognostic impact of incident left ventricular (LV) systolic dysfunction at the chronic phase of acute myocardial infarction (AMI).

**Materials and methods:**

Among 2,266 consecutive patients admitted for AMI, 1,330 patients with LV ejection fraction (LVEF) ≥ 40% during hospitalization who had LVEF data at 6 months after AMI were analyzed. Patients were divided into three subgroups based on LVEF at 6 months: reduced-LVEF (<40%), mid-range-LVEF (≥ 40% and < 50%) and preserved-LVEF (≥ 50%). Occurrence of a composite of hospitalization for heart failure or cardiovascular death after 6 months of AMI was the primary endpoint. The prognostic impact of LVEF at 6 months was assessed with a multivariate-adjusted Cox model.

**Results:**

Overall, the mean patient age was 67.5 ± 11.9 years, and LVEF during initial hospitalization was 59.4 ± 9.1%. The median (interquartile range) duration of follow-up was 3.0 (1.5–4.8) years, and the primary endpoint occurred in 35/1330 (2.6%) patients (13/69 [18.8%] in the reduced-LVEF, 9/265 [3.4%] in the mid-range-LVEF, and 13/996 [1.3%] in the preserved-LVEF category). The adjusted hazard ratio for the primary endpoint in the reduced-LVEF vs. mid-range-LVEF category and in the reduced-LVEF vs. preserved-LVEF category was 4.71 (95% confidence interval [CI], 1.83 to 12.13; p < 0.001) and 14.37 (95% CI, 5.38 to 38.36; p < 0.001), respectively.

**Conclusion:**

Incident LV systolic dysfunction at the chronic phase after AMI was significantly associated with long-term adverse outcomes. Even in AMI survivors without LV systolic dysfunction at the time of AMI, post-AMI reassessment and careful monitoring of LVEF are required to identify patients at risk.

## Introduction

In the primary percutaneous coronary intervention (PCI) era, an increased risk of late-onset heart failure (HF) and mortality as post-acute myocardial infarction (AMI) events is still an important clinical issue. Hence, a better risk stratification system to prevent those adverse events at the remote phase of AMI is of clinical importance. At the acute phase of acute coronary syndrome (ACS) including AMI, several established risk scores, such as TIMI (1) and GRACE (2), are universally used to predict prognosis. In addition, previous reports have suggested that some clinical manifestations, such as lack of reperfusion therapy (3), frailty (4), nutritional status (5), and a combination of multiple blood variables (6, 7) obtained at the acute-phase of AMI could predict adverse events after AMI. Thus, while the prognostic value of several clinical indicators in the acute phase of AMI has been established, predictors of long-term prognosis in the chronic phase of AMI have not yet been fully established.

Left ventricular ejection fraction (LVEF) is one of the most general indicators of left ventricular (LV) systolic function and is widely available in clinical settings. Existence of LV systolic dysfunction at the acute phase of AMI is well-known as a strong predictor for adverse prognosis after AMI (8). On the other hand, LVEF often changes dynamically through chronic LV remodeling after AMI (9), and this change in LVEF during the post-AMI period also has prognostic impact (10, 11). Thus, a chronic transition to LV systolic dysfunction can occur even in patients with AMI without systolic dysfunction at the acute phase of AMI, possibly adding to the risk of adverse events at the remote phase of AMI. However, the detailed clinical features and prognostic impact of incident reduced LVEF at the remote phase of AMI remain poorly elucidated. Focusing on newly developed LV systolic dysfunction at the chronic phase of AMI may help in understanding this clinical unmet need better. Herein, we sought to clarify the clinical features of incident LV systolic dysfunction at the remote phase of AMI and its prognostic impact among AMI survivors without LV systolic dysfunction at the acute phase of AMI.

## Materials and methods

### Design and population

This was a single-center, retrospective, observational study performed at Miyazaki Medical Association Hospital in Japan. A total of 2,266 consecutive patients admitted for AMI, with either ST-segment elevation myocardial infarction (STEMI) or non-ST-segment elevation myocardial infarction (NSTEMI), from February 2008 to January 2016 were eligible. Exclusion criteria were history of myocardial infarction, death within 6 months after AMI, admission due to worsening HF within 6 months after AMI, LVEF < 40% during index hospitalization, and no follow-up LVEF data at 6 months after AMI. According to LVEF at 6 months after AMI (within 1 month either side of 6 months), patients were divided into three subgroups: reduced-LVEF (< 40%), mid-range-LVEF (≥ 40% and < 50%), and preserved-LVEF (≥ 50%).

All procedures were followed in accordance with the ethical standards of the responsible committee on human experimentation (institutional and national) and with the Helsinki Declaration of 1964 and later revisions. All patients provided informed consent for both the procedure and the subsequent data collection and analysis for research purposes. Ethics approval was obtained from the Institutional Review Board of Miyazaki Medical Association Hospital (2019-23).

### Definition and diagnosis of ST-segment elevation myocardial infarction and non-ST-segment elevation myocardial infarction

Diagnosis of STEMI and NSTEMI, based on the 2007 universal definitions (12), was made by each cardiologist. STEMI and NSTEMI were defined as follows: for STEMI, patients had to have chest symptoms, ST-segment elevation in 2 contiguous leads or left bundle branch block, and an elevated biochemical marker of myocardial necrosis (high-sensitivity troponin T > 0.032 ng/mL or creatine phosphokinase [CPK] at least two times the upper limit of normal), whereas for NSTEMI, patients had to have chest symptoms, ST-segment depression or T-wave inversion in 2 contiguous leads, and an elevated biochemical marker of myocardial necrosis. The therapeutic strategies for AMI treatment depended on the practice of each individual cardiologist, but all treatments followed the guidelines set forth by the Japanese Circulation Society and the American College of Cardiology/American Heart Association for the diagnosis and treatment of AMI (13).

### Data collection and endpoints

Data collected included clinical characteristics and demographics during initial hospitalization, such as medical history, presenting signs and symptoms, results of blood tests, electrocardiography, cardiac procedures, and clinical outcomes. Transthoracic echocardiography was also carried out during index hospitalization and at around 6 months after AMI, and LVEF was estimated by the standard biplane Simpson method. In addition, all blood biomarkers were measured within 24 h after admission as acute phase data. Clinical follow-up was carried out through clinic visits, telephone calls, and records from hospital admissions.

The primary endpoint was a composite of hospitalization for HF or cardiovascular death occurred after 6 months of AMI. The diagnosis of HF was made based on the latest local guidelines, in which HF is diagnosed by the presence of at least one sign (rales, peripheral edema, ascites, or radiographic evidence of pulmonary congestion) and one symptom (dyspnea, orthopnea or edema), regardless of ejection fraction (14). Cardiovascular death was defined as the primary cause of death determined to be atherosclerotic cardiovascular disease, arrhythmia, heart failure, or sudden cardiac death. The secondary endpoints included the individual components of the primary endpoint and all-cause death.

### Statistical analysis

For continuous variables, normally distributed data are reported as the mean ± standard deviation; non-parametric data are reported as the median and interquartile range (IQR). For categorical variables, data are presented as count and percentage. Comparisons of continuous variables between groups were performed with the Wilcoxon-test or Kruskal Wallis tests, as appropriate. Comparisons of categorical variables were assessed with the chi-square or Fisher’s exact test, as appropriate. A paired sample t-test was used to compare LVEF at index hospitalization and 6 months after AMI. LVEF trajectories from index hospitalization for AMI to 6 months post-AMI were demonstrated using parallel plots. Clinical factors associated with LVEF category decline over the 6 months after AMI were assessed by logistic regression analysis adjusting for confounding factors (age, sex, STEMI, Killip class, culprit lesions (left anterior descending artery or left main trunk), use of mechanical support, maximum CPK [natural log-transformed], estimated glomerular filtration rate [eGFR], LVEF during index hospitalization and use of each medication at discharge; angiotensin-converting enzyme inhibitor [ACE-I] or angiotensin II receptor blocker [ARB] and β-blocker). The cumulative incidence of each endpoint was also calculated according to the Kaplan–Meier method, and the effects of LVEF 6 months after AMI on primary and secondary endpoints were determined with a multivariate Cox proportional hazards regression model adjusting for confounding factors (age, sex, STEMI, use of mechanical support, max CPK [natural log-transformed], eGFR, LVEF during index hospitalization and use of each medication at discharge; ACE-I or ARB and β-blocker). Time at risk was defined starting on the day of the 6-month LVEF measurement. A two-sided P value < 0.05 was considered statistically significant. All statistical analyses were performed with JMP^®^ 15 (SAS Institute Inc., Cary, NC, USA).

## Results

### Patient clinical characteristics during index hospitalization for acute myocardial infarction

**FIGURE 1:**
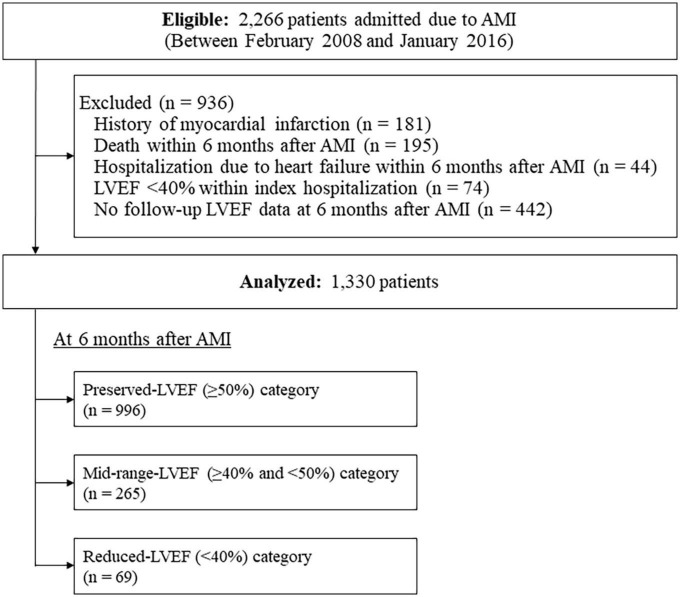
Flow diagram of the study cohort. AMI, acute myocardial infarction; LVEF, left ventricular ejection fraction.

Among a total of 2,266 consecutive patients eligible for this study, a total of 936 patients were excluded; thus, a total of 1,330 patients were analyzed (Figure 1). Their background characteristics, procedural information during index hospitalization, and medications at discharge are shown in Table 1. The mean patient age was 67.5 ± 11.9 years, with 74.1% being male. Electrocardiography revealed that 68.5% were STEMI, and almost all patients (95.1%) received primary revascularization (92.3% for PCI). Most patients received standard medical therapies after AMI at discharge.

**TABLE 1:** Patient background characteristics, procedural information, and medications at discharge.

Variable	Overall *n* = 1,330	LVEF category 6 months after AMI			*P*-value (among LVEF categories)
		Preserved-LVEF (≥ 50%) *n* = 996	Mid-range-LVEF (≥ 40% and < 50%) *n* = 265	Reduced-LVEF (< 40%) *n* = 69	
Age, years	67.5 ± 11.9	67.5 ± 11.9	67.7 ± 11.7	68.0 ± 11.7	0.928
Male	986 (74.1)	717 (72.0)	213 (80.4)	56 (81.2)	0.007
Body mass index, kg/m^2^	24.2 ± 3.6	24.2 ± 3.8	24.0 ± 3.4	24.1 ± 3.1	0.579
eGFR, mL/min/1.73 m^2^	68.0 ± 22.0	68.8 ± 21.3	66.5 ± 23.0	61.6 ± 26.7	0.025
**Medical history**					
Hypertension	928 (69.8)	713 (71.6)	165 (62.3)	50 (72.5)	0.014
Dyslipidemia	695 (52.3)	536 (53.8)	126 (47.6)	33 (47.8)	0.145
Diabetes mellitus	413 (31.1)	311 (31.2)	84 (31.7)	18 (26.1)	0.642
STEMI	911 (68.5)	649 (65.2)	213 (80.4)	49 (71.0)	< 0.001
NSTEMI	419 (31.5)	347 (34.8)	52 (19.6)	20 (29.0)	
Onset-to-admission time, min	180 (120-420)	180 (120-420)	180 (60-420)	240 (120-870)	0.108
Delayed arrival (≥ 48 h after onset)	35 (3.0)	27 (3.0)	7 (2.9)	1 (1.7)	0.804
Killip class ≥ 3	55 (4.2)	31 (3.1)	16 (6.0)	8 (11.6)	0.003
Culprit lesion					< 0.001
LMT	27 (2.0)	18 (1.8)	8 (3.0)	1 (1.4)	
LAD	566 (42.6)	396 (39.7)	132 (49.8)	38 (55.1)	
LCX	165 (12.4)	124 (12.8)	32 (12.1)	9 (13.0)	
RCA	483 (36.3)	383 (38.5)	86 (32.5)	14 (20.2)	
MVD and others	89 (6.7)	75 (8.3)	7 (2.6)	7 (10.1)	
Revascularization	1,266 (95.1)	949 (95.3)	253 (95.5)	64 (93.0)	0.676
PCI	1,227 (92.3)	922 (92.6)	242 (91.3)	63 (91.3)	0.765
CABG	39 (2.8)	27 (2.7)	11 (4.2)	1 (1.4)	0.481
Mechanical support	108 (8.1)	69 (6.9)	29 (10.9)	10 (14.5)	0.045
IABP	108 (8.1)	69 (6.9)	29 (10.9)	10 (14.5)	0.045
ECMO	8 (0.6)	6 (0.6)	2 (0.8)	0	0.629
Peak CPK, IU/L	1,417 (471-3,152)	1,124 (376-,367)	3,253 (1,159-5,319)	2,625 (981-5,764)	< 0.001
Hospital stay, days	15 (12-19)	14 (12–)	17 (14-22)	17 (14-23)	< 0.001
**Medication at discharge**					
ACE-I or ARB	933 (70.2)	719 (72.2)	175 (66.0)	39 (56.5)	0.007
β-blocker	619 (46.5)	442 (44.4)	137 (51.7)	40 (58.0)	0.016
MRA	108 (8.1)	55 (5.5)	42 (15.9)	11 (15.9)	< 0.001
Statin	1,165 (88.0)	886 (90.0)	223 (84.2)	60 (87.0)	0.113
Antiplatelet	1,300 (97.8)	979 (98.3)	255 (96.2)	66 (95.7)	0.181
LVEF during index hospitalization,%	59.4 ± 9.1	61.6 ± 8.4	53.6 ± 7.9	50.2 ± 7.7	< 0.001
LVEF at 6 months after AMI,%	49.0 ± 13.5	60.1 ± 6.5	45.5 ± 2.7	35.3 ± 4.0	< 0.001

Data for categorical variables given as number (%); data for continuous variables given as mean ± standard deviation for normal distribution or median (interquartile range) for skewed distribution. ACE-I, angiotensin-converting enzyme inhibitor; AMI, acute myocardial infarction; ARB, angiotensin II receptor blocker; CABG, coronary artery bypass grafting; CPK, creatine phosphokinase; ECMO, extracorporeal membrane oxygenation; eGFR, estimated glomerular filtration rate; IABP, intra-aortic balloon pumping; IU, international units; LAD, left anterior descending artery; LCX, left circumflex artery; LMT, left main trunk; LVEF, left ventricular ejection fraction; MRA, mineralocorticoid receptor antagonist; MVD, multi-vessel disease; NSTEMI, non-ST-elevation myocardial infarction; PCI, percutaneous coronary intervention; RCA, right coronary artery; STEMI, ST-elevation myocardial infarction.

### Left ventricular ejection fraction trajectories from index hospitalization for acute myocardial infarction to 6 months post-acute myocardial infarction

**FIGURE 2:**
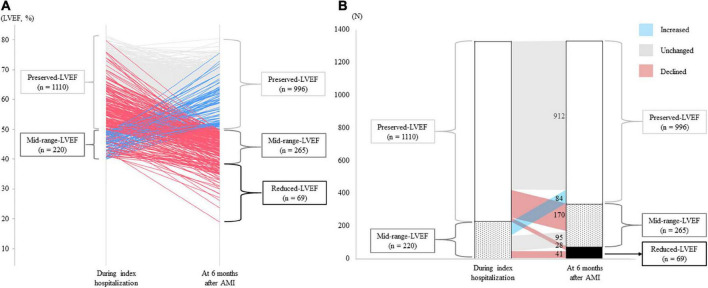
LVEF trajectories from index hospitalization for AMI to 6 months post-AMI. **(A)** Individual trajectories of LVEF over the 6 months after AMI. Blue, gray, red lines indicate patients with increased, unchanged, and declined LVEF, respectively. **(B)** Changes in LVEF categories. The numbers in the figure indicate the number of patients whose LVEF category changed or did not change 6 months after AMI. AMI, acute myocardial infarction; LVEF, left ventricular ejection fraction.

Overall, mean LVEF during index hospitalization and 6 months after AMI was 59.4 ± 9.1% and 49.0 ± 13.5% (p < 0.001), respectively (Table 1). The detailed trajectories of LVEF from index hospitalization to 6 months after AMI are shown in Figure 2A. A total of 69 patients (28/1,110 [2.5%] initially in the preserved-LVEF and 41/220 [18.6%] initially in the mid-range-LVEF categories) newly developed reduced-LVEF at 6 months, and a total of 170/1,100 (15.5%) patients initially in the preserved-LVEF category declined to the mid-range-LVEF category at 6 months (Figure 2B). Conversely, a total of 84/220 (38.2%) patients initially in the mid-range-LVEF category climbed from that to the preserved-LVEF category at 6 months. The LVEF categories in the other patients remained unchanged at 6 months after AMI.

Detailed clinical information at the time of index hospitalization in the three subgroups stratified by LVEF category at 6 months after AMI is also provided in Table 1. The subgroups with mid-range- and reduced-LVEF at 6 months after discharge, relative to the preserved-LVEF subgroup, were more likely to have a higher proportion of males, a lower eGFR, and a more severe clinical course of AMI.

The multivariate logistic regression analysis revealed that among 239 patients whose LVEF category declined at 6 months after AMI, male sex and peak CPK were independently associated with the decline, while LVEF during index hospitalization and use of ACE-I or ARB at discharge were inversely associated with a decline (Table 2). Among those factors, the use of ACE-I or ARB at discharge was solely an independent negative predictor of a two-step LVEF category decline (Table 3). Notably, ACE-I or ARB therapy was not an independent predictor of improved LVEF at 6 months, but female sex and LVEF during hospitalization were found to be associated (Supplementary Table 1).

**TABLE 2:** Logistic regression analysis to identify clinical factors associated with LVEF category decline over the 6 months after AMI.

Variable	Odds ratio	95% CI	*P*-value
Age, per 1 year	1.01	0.99-1.03	0.075
Male	1.76	1.19-2.63	0.005
STEMI	0.73	0.49-1.10	0.135
Killip class ≥ 3	1.07	0.51-2.23	0.853
Culprit lesion: LAD or LMT	1.27	0.93-1.74	0.131
Use of mechanical support	0.90	0.51-1.60	0.735
Peak CPK, ln U/L	1.64	1.40-1.93	< 0.001
eGFR, per 1 mL/min/1.73 m^2^	0.99	0.99-1.00	0.205
LVEF during index hospitalization, per 1%	0.96	0.94-0.98	< 0.001
Use of ACE-I or ARB at discharge	0.62	0.44-0.86	0.004
Use of β-blocker at discharge	1.10	0.80-1.51	0.548

CI, confidence interval; other abbreviations, see Table 1.

**TABLE 3:** Logistic regression analysis to identify clinical factors associated with a decline in LVEF category over the 6 months after AMI.

Variable	One-step LVEF category decline*			Two-step LVEF category decline**		
	Odds ratio	95% CI	*P*-value	Odds ratio	95% CI	*P*-value
Age, per 1 year	1.01	0.99-1.03	0.071	1.00	0.97-1.04	0.897
Male	1.77	1.16-2.69	0.008	1.42	0.51-4.00	0.503
STEMI	0.82	0.53-1.26	0.359	0.47	0.18-1.23	0.125
Killip class ≥ 3	1.09	0.50-2.34	0.833	0.93	0.18-4.81	0.935
Culprit lesion: LAD or LMT	1.23	0.88-1.70	0.222	1.39	0.63-3.08	0.421
Use of mechanical support during procedures	0.74	0.40-1.36	0.328	2.28	0.76-6.88	0.142
Peak CPK, ln U/L	1.63	1.37-1.93	< 0.001	1.45	0.98-2.16	0.057
eGFR, per 1 mL/min/1.73 m^2^	0.99	0.99-1.00	0.158	0.99	0.98-1.02	0.843
LVEF during index hospitalization, per 1%	0.96	0.94-0.98	< 0.001	0.99	0.95-1.04	0.696
Use of ACE-I or ARB at discharge	0.70	0.49-0.99	0.044	0.39	0.17-0.86	0.020
Use of β-blocker at discharge	1.07	0.77-1.49	0.697	1.23	0.55-2.76	0.613

*For 211 patients whose LVEF declined from preserved- to mid-range-LVEF or mid-range- to reduced-LVEF at 6 months after AMI. **For 28 patients whose LVEF declined from preserved- to reduced-LVEF at 6 months after AMI. Abbreviations, see Tables 1, 2.

### Clinical endpoints

**FIGURE 3:**
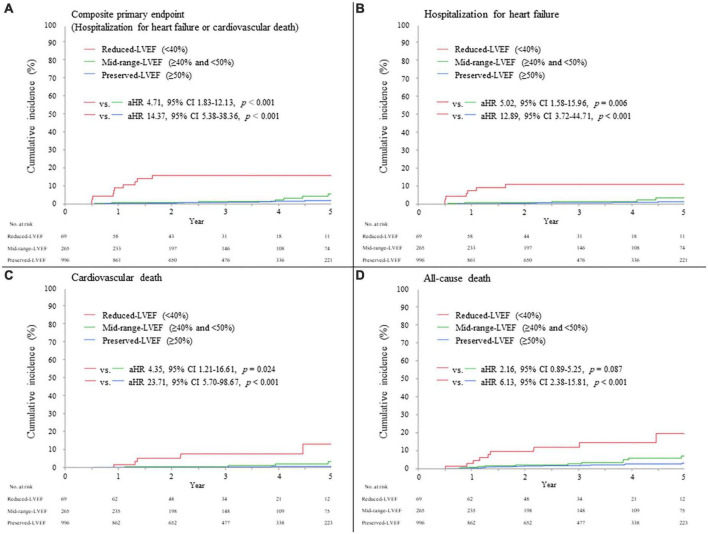
Clinical events during follow-up, according to LVEF category 6 months after AMI. **(A)** Composite primary endpoint (hospitalization for heart failure or cardiovascular death). **(B)** Hospitalization for heart failure. **(C)** Cardiovascular death. **(D)** All-cause death. The hazard ratio was adjusted by age, sex, STEMI, maximum creatine phosphokinase (natural log-transformed), LVEF during index hospitalization, eGFR, use of mechanical support, and use of each medication (ACE-I, ARB, and β-blockers) at discharge. ACE-I, angiotensin-converting enzyme inhibitor; AMI, acute myocardial infarction; aHR, adjusted hazard ratio; ARB, angiotensin II receptor blocker; CI, confidence interval; LVEF, left ventricular ejection fraction; STEMI, ST-segment elevation myocardial infarction.

The median (interquartile range) duration of follow-up 6 months after AMI was 3.0 (1.5–4.8) years. Overall, the primary composite endpoint of hospitalization for HF or cardiovascular death occurred in 35/1,330 (2.6%) patients (13/996 [1.3%] in the preserved-LVEF, 9/265 [3.4%] in the mid-range-LVEF, and 13/69 [18.8%] in the reduced-LVEF categories, Log-rank p < 0.001); individual components of the primary composite endpoint occurred in 21/1,330 (1.6%) patients for hospitalization for HF and 19/1,330 (1.4%) patients for cardiovascular death (Table 4). The adjusted hazard ratio (HR) for the primary endpoint in the reduced-LVEF vs. mid-range-LVEF categories and in the reduced-LVEF vs. preserved-LVEF categories was 4.71 (95% confidence interval [CI], 1.83 to 12.13; p < 0.001) and 14.37 (95% CI, 5.38 to 38.36; p < 0.001), respectively (Figure 3A). These were almost consistent across the individual components of primary composite endpoint; hospitalization for HF and cardiovascular death (Figures 3B,C). All-cause death occurred in 50/1,330 (3.8%) patients in the overall cohort (23/996 [2.3%] in the preserved-LVEF, 15/265 [5.7%] in the mid-range-LVEF, and 12/69 [17.4%] in the reduced-LVEF categories, Log-rank p < 0.001). The adjusted HR for all-cause death in the reduced-LVEF vs. mid-range-LVEF categories and in the reduced-LVEF vs. preserved-LVEF categories was 2.16 (95% CI, 0.89 to 5.25; p = 0.087) and 6.13 (95% CI, 2.38 to 15.81; p < 0.001), respectively (Figure 3D).

**TABLE 4:** Clinical events.

Outcomes	Overall *n* = 1,330	LVEF category 6 months after AMI			*P*-value (Log-rank)
		Preserved-LVEF (≥ 50%) n = 996	Mid-range-LVEF (≥ 40% and < 50%) n = 265	Reduced-LVEF (< 40%) n = 69	
Composite outcome	35 (2.6)	13 (1.3)	9 (3.4)	13 (18.8)	< 0.001
Hospitalization for heart failure	21 (1.6)	7 (0.7)	6 (2.3)	8 (11.6)	< 0.001
Cardiac death	19 (1.4)	6 (0.6)	5 (1.9)	8 (11.6)	< 0.001
All-cause death	50 (3.8)	23 (2.3)	15 (5.7)	12 (17.4)	< 0.001

Data are shown as number (%). Abbreviations, see Table 1.

## Discussion

This is the report to demonstrate the clinical features of chronic transit of LVEF and incident LV systolic dysfunction at the chronic phase of AMI and its long-term prognostic impact in AMI survivors. Our findings underscore the clinical importance of monitoring LVEF through the post-AMI phases, even in survivors without LV systolic dysfunction at the acute phase of AMI.

In the past two decades, widespread technical innovations in primary coronary revascularization for AMI have dramatically increased the number of AMI survivors. Accordingly, an increased risk of HF and mortality at the post-AMI phase has become an emerging clinical issue of concern, urgently requiring accurate and reliable risk stratification to predict such remote-phase adverse events (15). Traditionally, some risk prediction models, such as GRACE and TIMI, both of which consist of indicators obtained at the acute phase of AMI, have been universally used to predict the prognosis of patients with AMI (1, 2). On the other hand, such indicators obtained during the acute phase are highly variable, depending on the individual clinical situation and the course of treatment during the acute to post-AMI phases. Therefore, risk stratification based on the clinical index obtained at the chronic phase and its change from the acute to the chronic phase may contribute to the improvement of a longer-term prognostic ability for patients who have experienced AMI. However, there are few studies on long-term prognostic prediction based on the clinical data obtained at the chronic phase.

LVEF is an established indicator of LV systolic function, and LV systolic dysfunction (reduced LVEF) at the acute phase of AMI is also well-recognized as an independent predictor of adverse outcomes (16, 17). However, it is still clinically controversial whether the sole use of LVEF measured only at the acute phase is sufficient to predict the long-term prognosis (18). Moreover, substantial patients often exhibit mildly reduced- or preserved-LVEF immediately following AMI, and the majority of adverse events after AMI develop in that patient population without overt LV dysfunction at the acute-phase (19, 20). Therefore, it is clinically important to assess the mechanism by which patients without LV systolic dysfunction at the acute phase of AMI develop adverse events at the remote phase. In this context, we hypothesized that LVEF at the chronic phase would be a predictor of subsequent events in survivors with preserved LVEF at the acute phase, and then evaluated the long-term prognostic impact according to LVEF at the chronic phase and its trajectory from the acute to the chronic phases.

After the onset of AMI, immediate coronary revascularization and subsequent optimal medical therapies help to prevent adverse LV remodeling and thereby improve LV systolic function. To date, several studies of subjects with reduced LVEF at the time of AMI have demonstrated that chronic LVEF recovery was associated with better outcomes in comparison with survivors without LVEF recovery (11, 21). Chew et al. followed patients with only reduced EF during the acute phase of myocardial infarction (22). They demonstrated that the absence of LVEF recovery is associated with an increased risk of death. This suggests that patterns of chronic change in LVEF following AMI can further discriminate AMI survivors who are at increased risk of death. However, it is uncertain how the LVEF status in patients with preserved LVEF at the time of AMI transitions over time. Further, the clinical characteristics of the trajectory pattern of LVEF are unknown. Compared to a study in which AMI patients with preserved LVEF in the acute phase were excluded, the present study excluded AMI patients with reduced LVEF in the acute phase, and focused on chronic changes in AMI patients with preserved LVEF.

Our findings underscore that careful post-AMI reassessments are required to monitor the LVEF trajectory and identify potential patients who need additional medical and/or device therapies, even in patients with preserved LVEF at the time of AMI. However, despite guideline-directed recommendations (23, 24), previous studies have shown that the frequency of post-AMI LVEF reassessment was relatively low in patients with LV systolic dysfunction at the time of AMI (25, 26). A recent cohort study from Canada also demonstrated that approximately 1 in 3 patients with mildly reduced LVEF following AMI did not undergo LVEF reassessment within 6 months after AMI (27). The low frequency of post-AMI LVEF reassessment indicates a missed opportunity for appropriate care, especially for LV systolic dysfunction. Importantly, few data on the rate of post-AMI LVEF reassessment in patients with preserved LVEF at the time of AMI are currently available, and it is likely even less frequent for such patients. In addition, given our findings that incident LV systolic dysfunction 6 months after AMI was associated with poor outcomes, improvements in the quality of post-AMI management are urgently needed, including post-AMI LVEF reassessment, irrespective of LVEF status at index AMI.

The development of HF remains a major issue in AMI patients. Several clinical features, such as elevated levels of natriuretic peptides and a clinically severe AMI disease course are known to be risk factors (28). Delayed arrival causes a delay in reperfusion therapy, which often results in a larger infarct size and increased risk of HF (29). In the present study cohort, the frequency of delayed arrival after AMI onset in the subgroup with EF ≥ 40% and < 50% at 6 months after AMI was higher than that in the subgroup with reduced LVEF (< 40%) at 6 months. This might be associated with the higher peak CPK levels in the former subgroup. Several previous reports have addressed the potential risk predictors for the occurrence of early- and late-onset HF after AMI. However, the factors were diverse (30), and no specific factor was identified for either early- or late-onset HF (31, 32). In terms of echocardiographic parameters, there have been also several reports on the evaluation of chronic LVEF at a single point in time and the development of HF (33). However, LVEF dynamics and the assessment of late-onset HF according to their trajectories in the remote phase of AMI have not yet been fully studied.

In the present study, we found that the prevalence of incident LV systolic dysfunction 6 months after AMI was 5.2% among AMI survivors without LV systolic dysfunction at the time of AMI, and such patients were associated with poor long-term prognosis compared to subjects without it. This indicates the clinical need for early identification of patients at risk for LVEF decline during the chronic phase of AMI. In this context, male sex, peak CPK level, LVEF at the time of AMI, and use of ACE-I or ARB at discharge were independent predictors of LVEF decline 6 months after AMI. In particular, the use of ACE-I or ARB at discharge was solely an independent negative predictor of a two-step decline in LVEF category. In the previous PREAMI (Perindopril and Remodeling in Elderly With Acute Myocardial Infarction) study, Ferrari et al. also demonstrated that 12 months of ACE-I perindopril therapy rescued adverse LV remodeling in elderly patients with a LVEF 40% or more following AMI (34). This is likely comparable to our findings from multivariate regression analyses. Although no relationship between the prevention of adverse LV remodeling and better clinical outcomes was observed in that study, the short observation period (12 months) might have affected the outcome. Compared to that study, the strength of our study is that we reassessed the LVEF 6 months after AMI and then had a longer follow-up period (median 3 years). On the other hand, Park et al. reported that the dose of ARB had no impact on LV remodeling in patients with mid-range LVEF following AMI (35). Taken together, these findings suggest the importance of administering ACE-I or ARB, even in the absence of LV systolic dysfunction immediately after AMI.

## Limitations

Some limitations must be taken into account. First, this was a retrospective, observational study carried out in a relatively small number of subjects at a single center, which limits the generalizability of our findings. It should also be noted that primary coronary revascularization and subsequent oral medication delivery were performed based on the latest local treatment guidelines. However, decision-making regarding hospitalization for HF was the choice of each physician; therefore, relevant endpoints were partly based on physicians’ subjective judgment. Second, because the study cohort included only survivors 6 months after AMI to collect remote data on LVEF, a potential selection bias should be noted. Accordingly, the occurrence of composite clinical events (hospitalization for HF and cardiovascular death) was low (2.3%) during the follow-up duration. Additionally, patients with reduced LVEF at 6 months already had worsening of some clinical indicators, such as lower EF and eGFR levels and a higher proportion of patients with Killip class ≥ 3, at index hospitalization, and this patient subgroup was therefore not entirely representative of the overall cohort. Third, our study cohort included both STEMI and NSTEMI, with two-thirds of subjects showing STEMI; this rate is higher relative to a contemporary cohort for AMI in Japan (36, 37). The prognostic impact of LVEF at the chronic phase was not investigated separately between STEMI and NSTEMI due to the limited small sample size in our cohort. Fourth, the rate of prescribing optimal drug therapy after AMI was lower than expected in our cohort. Specifically, a relatively small proportion of subjects was treated with β-blockers due mainly to tolerability, and this was similar to previous reports in Japan (36, 37). However, we cannot exclude the possibility that such incomplete implementation of optimal medical therapy after AMI might have affected the patients’ prognosis and our findings. Finally, the present analysis did not account for any clinical information that may have affected long-term prognosis in survivors of AMI, including biomarkers, at the chronic phase other than LVEF. Therefore, further studies are needed to investigate the clinical parameters related to LVEF dynamics and assess the prognostic relationships between their trajectories in the remote phase of AMI.

## Conclusion

Our findings suggest that incident LV systolic dysfunction at the chronic phase after AMI was significantly associated with long-term adverse outcomes. Therefore, even in AMI survivors without LV systolic dysfunction at the time of AMI, post-AMI reassessment and careful monitoring of LVEF are required to identify patients at risk. Patient with risk factor, such as male sex and higher peak CPK should be followed more carefully.

## Data availability statement

The raw data supporting the conclusions of this article will be made available by the authors, without undue reservation.

## Ethics statement

The studies involving human participants were reviewed and approved by The Institutional Review Board of Miyazaki Medical Association Hospital. The patients/participants provided their written informed consent to participate in this study.

## Author contributions

GY and AT designed the research, project conception, development of overall research plan, study oversight, and wrote the manuscript. GY, KNi, NW, YS, and MN conducted the research, hands-on conduct of the experiments, and data collection. GY, AT, and AK analyzed the data or performed statistical analysis. MN and KNo revised the manuscript. GY and AT had primary responsibility for the final content. All authors read and approved the final manuscript.
