# Supplementary material for: Prognostic impact of incident left ventricular systolic dysfunction after myocardial infarction
Source: Front Cardiovasc Med. 2022 Sep 29;9:1009691. doi: 10.3389/fcvm.2022.1009691 (PMC9557083; doi:10.3389/fcvm.2022.1009691)
Supplement: Supplementary file 1 [file Table_1.DOCX]

|  | **LVEF category decline improved*** | | |
| --- | --- | --- | --- |
|  | **Odds ratio** | **95% CI** | ***P*-value** |
| Age, per 1 year | 1.01 | 0.98-1.04 | 0.540 |
| Male | 0.48 | 0.25-0.92 | 0.028 |
| Female | 2.06 | 1.08-3.93 | 0.028 |
| STEMI | 0.69 | 0.32-1.52 | 0.365 |
| Killip class ≥3 | 1.16 | 0.41-3.26 | 0.781 |
| Culprit lesion: LAD or LMT | 1.16 | 0.63-2.12 | 0.641 |
| Use of mechanical support during procedures | 1.02 | 0.40-2.57 | 0.968 |
| Peak CPK, ln U/L | 0.90 | 0.70-1.18 | 0.456 |
| eGFR, per 1 mL/min/1.73 m^2^ | 1.01 | 0.99-1.02 | 0.410 |
| LVEF during index hospitalization, per 1% | 0.74 | 0.70-0.79 | < 0.001 |
| Use of ACE-I or ARB at discharge | 1.77 | 0.89-3.53 | 0.102 |
| Use of β-blocker at discharge | 0.69 | 0.38-1.27 | 0.238 |

**Supplementary Table 1: Logistic regression analysis to identify clinical factors associated with an improved in LVEF category over the 6 months after AMI**

*For 84 patients whose LVEF improved from mid-range- to preserved-LVEF at 6 months after AMI.

ACE-I, angiotensin-converting enzyme inhibitor; AMI, acute myocardial infarction; ARB, angiotensin II receptor blocker; CI, confidence interval; CPK, creatine phosphokinase; eGFR, estimated glomerular filtration rate; LAD, left anterior descending artery; LMT, left main trunk; LVEF, left ventricular ejection fraction; STEMI, ST-elevation myocardial infarction.
